# ShadowVIMP: permutation-based multiple testing-controlled variable selection

**DOI:** 10.1186/s12859-026-06412-4

**Published:** 2026-05-02

**Authors:** Tim Müller, Roman Hornung, Silke Szymczak, Hannes Buchner

**Affiliations:** 1 Staburo GmbH, Aschauer Straße 26a, 81549 Munich, Bavaria Germany; 2 Institute for Medical Information Processing, Biometry and Epidemiology, Faculty of Medicine, Ludwig-Maximilians-Universität München, Marchioninistraße 15, 81377 Munich, Bavaria Germany; 3 Munich Center for Machine Learning (MCML), Munich, Germany; 4 Institute of Medical Biometry and Statistics, University of Lübeck, Ratzeburger Allee 160, 23562 Lübeck, Germany

**Keywords:** Random forest, Variable importance, Variable selection, High-dimensional, Multiple testing correction

## Abstract

****Background**:**

Identifying relevant biomarkers is critical in clinical research and precision medicine, particularly when analysing high-dimensional data. Random forests (RFs) are promising for such settings due to their flexibility, ease of use, and their ability to handle data sets with more variables than samples. RFs assess the importance of each variable in predicting the outcome using variable importance (VIMP) scores. However, since the distribution of VIMP scores is intricate, standard statistical testing and multiple testing adjustments for variable selection are challenging.

****Methods**:**

We propose shadowVIMP, a novel method for multiple testing-controlled variable selection, based on an approach similar to permutation testing. It generates permuted counterparts for each variable and compares their VIMPs with those of the original variables over multiple iterations to calculate *p*-values. Unlike conventional permutation testing, shadowVIMP preserves the correlation structure between variables, mitigating biases caused by the over-selection of correlated variables in RFs. We evaluated shadowVIMP against three competing RF variable selection approaches using simulation designs previously employed in studies considering VIMPs and variable selection for RFs. These designs included high- and low-dimensional data, as well as correlated and categorical variables. For illustration, we also applied the method to a real-world example on Alzheimer’s disease.

****Conclusions**:**

Our results showed that, compared to competing approaches, shadowVIMP offers advantages in high-dimensional settings, improving sensitivity while enabling multiple testing-adjusted results. Additionally, it demonstrated robustness against VIMP biases induced by correlated and categorical variables when using permutation-based VIMP. The method can be used to annotate standard VIMP plots, visually presenting selected variable sets based on different types of multiple testing adjustments and significance levels. Overall, shadowVIMP is a promising approach for providing multiple testing-adjusted variable selection while explicitly addressing known biases of RF’s permutation-based VIMP measure. The shadowVIMP method is implemented in an R package shadowVIMP, which is available on CRAN.

## Background

Random forests (RF), introduced by Breiman [1], have become a widely used tool for classification and regression in various research domains. They consist of multiple decision trees built from bootstrap samples or subsamples of the original data, offering flexibility through their ability to handle non-linear effects and interactions [2]. The non-parametric approach is particularly valuable in high-dimensional data settings, where the number of variables exceeds the number of observations [3, 4]. Furthermore, RFs have a long track record of good accuracy in prediction tasks. For example, in a large-scale benchmark experiment employing 243 data sets, RFs outperformed logistic regression in 69% of the cases [5]. A fast implementation of RFs is available in the R package ranger [6].

In many cases, creating a well-performing prediction model is only of secondary interest; a more critical goal is to identify the variables that are important for prediction [4]. Clinical researchers, for example, are often less concerned with implementing a predictive model in practice and more interested in discovering the factors that contribute to it [7].

The RF algorithm includes several built-in measures to assess a variable’s influence on the prediction. This work focuses on the permutation variable importance measure (VIMP), which quantifies the change in the RF’s accuracy when a variable is permuted in the samples of a test set, effectively breaking its association with the outcome. If the change in accuracy is substantial, it implies that the variable plays a crucial role in the prediction. Conversely, if the change is near zero or negative, this suggests that the variable has an importance comparable to that of uninformative noise variables.

**Fig. 1:**
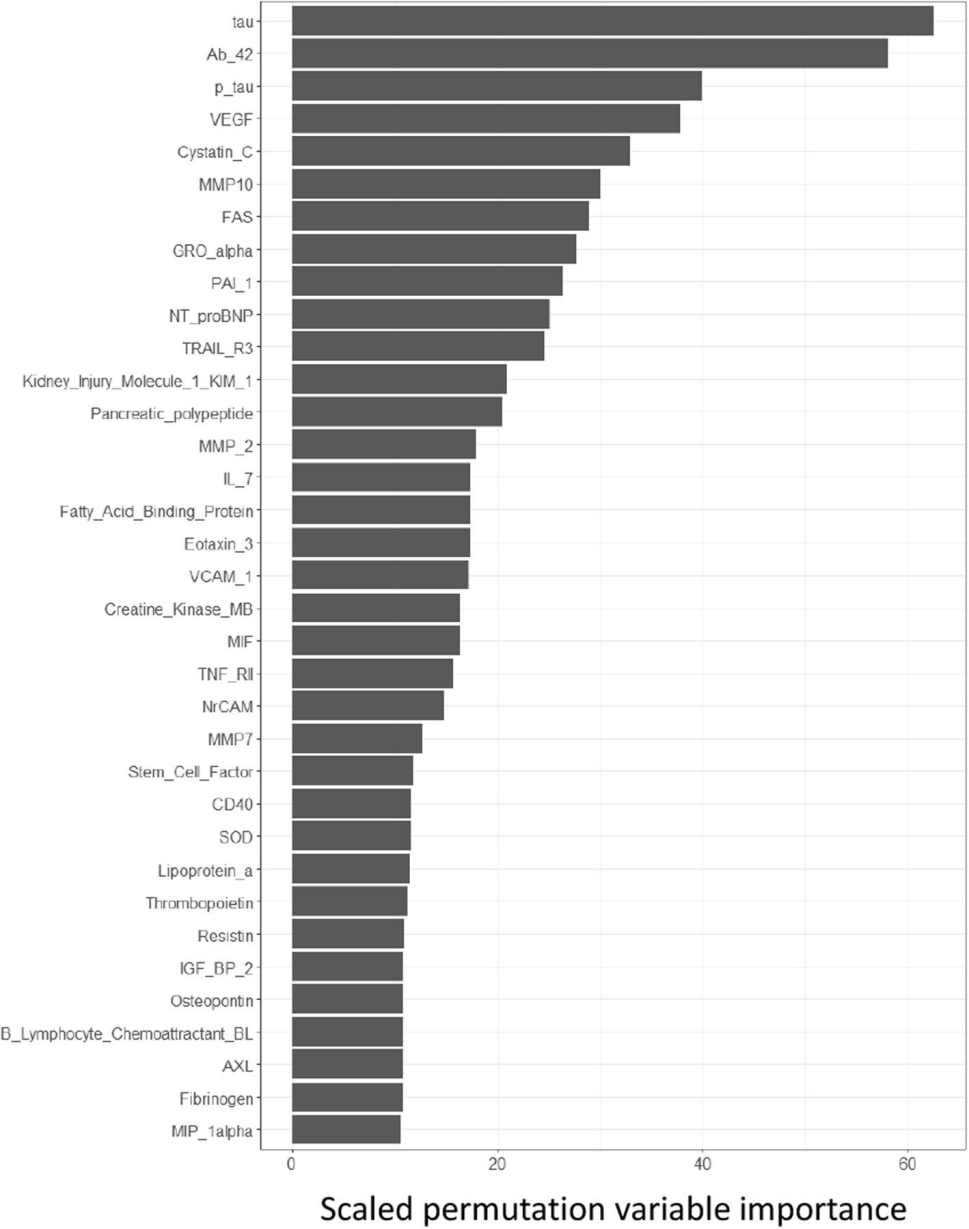
Illustration of RF VIMPs on a modified version of the data from Craig-Schapiro et al. [8], as provided in the R package AppliedPredictiveModeling [9]. Only the top 35 biomarkers, out of a total of 130, are shown

A limitation of the VIMP measure, from an interpretational perspective, is that while it yields the importance scores on a continuous scale, it only offers a ranking of variables in terms of their influence on the prediction. More specifically, there is no established theoretical threshold for determining when the importance of a variable is large enough to conclude that the variable is informative. As a motivating example, consider the study by Craig-Schapiro et al. [8] in which the RF method (among other techniques) was used to identify new biomarker candidates to improve established diagnostic and prognostic prediction models for Alzheimer’s disease. Figure 1 displays the VIMPs of the highest scoring variables of a single RF run on the data made available in the R package AppliedPredictiveModeling [9]. To make a final selection, [8] chose the cut-off point after the 15 most important biomarkers. The practice of setting the cutoff point at some number of highest-ranking variables is common, as pointed out by Janitza et al. [2]. However, they further note that this approach is problematic, as one would always select some variables as informative even if all variables in the data are uninformative for the outcome. Conversely, if the threshold is set too conservatively, there is a risk of overlooking genuinely informative variables.

What is often needed in RF-based VIMP analysis is a statistical testing procedure to determine whether the observed importance scores are unlikely under a null hypothesis of no association [2]. The key challenge is establishing an appropriate null distribution, as the theoretical distribution of RF VIMPs is intricate. An early attempt, the z-score test by Kuhn et al. [10], was found to have undesirable statistical properties [2, 11].

To address this, [12] proposed permuting one variable at a time while preserving the associations of all others with the outcome. This approach generates a null distribution that more closely reflects the intuitive notion of testing the significance of a single variable. However, it is computationally intensive when considering larger data sets, as separate permutations and RF runs are required for each variable. A subsequent refinement introduced sequential testing to the method to reduce runtime [13] implemented in the R package rfvimptest.

The Boruta algorithm [14] offers a more runtime-efficient permutation strategy by appending permuted ’shadow’ variables, copies of the original variables with their association to the outcome removed, to the data set. The VIMP of each variable is then compared with the maximum VIMP scored among the shadow variables. Although Boruta generally achieves a good balance between true and false positives [4, 15], it does not produce *p*-values. This limits both the interpretability of its results and the flexibility to control error rates using standard significance thresholds.

Another approach, recommended alongside Boruta by Degenhardt et al. [4] for high-dimensional data, is the Vita method proposed by Strobl et al. [2]. Unlike permutation-based methods, Vita approximates the null distribution by mirroring the VIMPs of variables with non-positive scores at the origin, under the assumption that such variables are unrelated to the outcome and thus represent one side of the underlying null distribution. By avoiding permutations, Vita is computationally efficient and particularly well-suited for high-dimensional settings, where many uninformative variables are typically present. However, the granularity of *p*-values is inherently constrained by the number of these variables, raising concerns about the suitability of the method for stringent multiple testing corrections, which remain relatively unexplored.

**Contributions** In this work, we propose a novel approach–shadowVIMP–designed to support variable selection in both low- and high-dimensional settings, while producing *p*-values suitable for standard multiple testing correction procedures. The method builds on the concept introduced by the Boruta algorithm: extending the data set with shadow variables, which serve as permuted counterparts to the original predictors. The VIMP values obtained from the original variables are then compared to those from the shadow variables to assess statistical significance.

However, shadowVIMP introduces a different permutation scheme for generating the shadow variables. Rather than independently permuting each variable, the method replicates the correlation structure of the original predictors within the shadow variables. In light of the findings by Nicodemus et al. [16] on the impact of correlations among variables when using permutation-based VIMP measures, this design enables comparisons that are not confounded by the correlation structure. Row-wise (or group-wise) permutation has been discussed previously in multivariate contexts–see, [17] for a comprehensive review–but here we apply it specifically to the construction of shadow variables.

Importantly, shadowVIMP outputs *p*-values that are compatible with widely used multiple testing correction methods such as FDR and FWER adjustments, an essential requirement in high-dimensional analyses. We conducted an extensive simulation study comparing shadowVIMP with existing methods, including Vita, rfvimptest, and Boruta, using five simulation designs previously employed in research on VIMP and variable selection for RFs. The results indicate that shadowVIMP maintains nominal significance levels while enabling multiple testing correction. Moreover, it demonstrates equal or superior sensitivity in detecting relevant variables and shows resilience in scenarios involving correlated or categorical predictors, avoiding previously reported over-selection issues [3, 16].

To illustrate its practical utility, we provide an example application in which shadowVIMP results are directly integrated into standard RF VIMP plots. These visualizations highlight variables with importance scores unlikely to have occurred by chance and offer insights into variable selection decisions under various error control strategies, such as FDR or FWER.

## Methods

### Notation

We denote the data set of sample size *n* by $D = \left[ y, X \right]$, where the outcome vector is given by $y \in \mathbb {R}^{n}$ in the case of regression, and $y \in \left( 1,2,...,C\right) ^{n}$ in the case of classification with *C* classes. The predictor matrix is defined as $X \in \mathbb {R}^{n \times p} = \left( x_1, x_2, \dots , x_p \right)$, where each column vector $x_j = \left( x^{(1)}_j, x^{(2)}_j, \dots , x^{(n)}_j \right)$ contains the values of the *j*-th predictor variable across all samples, for $j = 1, \dots , p$. Consequently, a single observation (that is, one sample) is denoted by $x^{(i)} = \left( x_1^{(i)}, x_2^{(i)}, \dots , x_p^{(i)} \right)$, for $i = 1, \dots , n$.

Let $\pi : S \rightarrow S$ be a permutation function acting on a set $S = \left( s_{(1)}, s_{(2)}, \dots , s_{(n)} \right)$, such that $\pi$ rearranges all elements of *S*. A particular permutation $\vartheta$ from the set of all possible permutations $\Theta$ can be represented as: $\pi (S|{\Theta = \vartheta }) = \left( s_{\pi (1|{\vartheta })}, s_{\pi (2|{\vartheta })},..., s_{\pi (n|{\vartheta })} \right)$. We consider three distinct permutation schemes applied to the predictor matrix *X*:

1 $$\begin{aligned}&X_{\pi (x_j|\vartheta )} := \left( x_1, x_2, ..., \pi (x_j|\vartheta ), ... , x_p \right) \end{aligned}$$

2 $$\begin{aligned}&{X}_{\pi (x_1,..., x_p|\vartheta _1,..., \vartheta _p)} := \left( \pi (x_1|\vartheta _1), \pi (x_2|\vartheta _2), ... , \pi (x_p|\vartheta _p) \right) \end{aligned}$$

3 $$\begin{aligned}&{X}_{\pi (x_1,..., x_p|\vartheta )} := \left( \pi (x_1|\vartheta ), \pi (x_2|\vartheta ), ... , \pi (x_p|\vartheta ) \right) \end{aligned}$$

Equation (1) represents the predictor matrix with only the *j*-th variable permuted. Equation (2) permutes each variable independently using distinct permutations, similar to the approach used in Boruta to generate shadow variables. Equation (3) applies the same permutation to all variables simultaneously, effectively permuting the rows (that is, the samples) of the predictor matrix *X*.

### Random forest based variable importance and sources of bias

The RF algorithm constructs an ensemble of decision trees, each trained on a bootstrap sample (sampling with replacement) or a subsample (sampling without replacement) of the original data. Each tree is built by recursively partitioning the data using binary splits. At each split, a variable is selected from a random subset of size mtry, and the best split is determined: in regression, by minimizing the within-node variance; in classification, by minimizing impurity measures such as Gini impurity or entropy.

A prediction for a new sample is made by passing it through each tree to reach a terminal node. In regression, the prediction of a tree corresponds to the mean outcome of training observations in the terminal node. In classification, predictions are either class probabilities (based on class proportions) or a single class label (majority vote). The final RF prediction is obtained by aggregating the predictions of all trees. For the remainder of this section, we focus on the regression case. The classification case is analogous for all relevant aspects and is omitted for brevity.

RF includes an internal estimate of prediction error using out-of-bag (OOB) observations - samples not included in the bootstrap or subsample used to construct a given tree. For each observation, predictions are made using only the trees for which the observation was OOB, and the average prediction error over all observations yields the OOB error estimate.

The use of OOB observations in RF facilitates the calculation of the permutation variable importance (VIMP), a measure intended to quantify the predictive contribution of individual variables. Among various VIMP measures, we focus on permutation VIMP, which assesses the change in prediction error when a variable’s values are permuted.

Permuting a variable disrupts its association with the outcome, thereby effectively randomizing all splits using that variable as the splitting variable. A substantial increase in prediction error after permutation suggests that the variable is important for prediction, wheresas a minor change indicates limited relevance for the prediction.

We present the regression case here; the classification case is analogous. Let $B_l$ denote the set of OOB samples for tree *l*. For the permutation VIMP of variable $x_j$, the contribution of tree *l* is given by:

4 $$\begin{aligned} VI^{(l)}(x_j) = \frac{1}{|B_l|} \sum _{i \in B_l} \left( (\hat{y}^{(i)} | X_{\pi ^{B_l}(x_j|\vartheta )}, l) - y^{(i)} \right) ^2 - \frac{1}{|B_l|} \sum _{i \in B_l} \left( (\hat{y}^{(i)} | X, l) - y^{(i)} \right) ^2. \end{aligned}$$

Here, $\pi ^{B_l}(x_j| \vartheta )$ denotes a permutation of the feature $x_j$ applied only to the observations $i \in B_l$, with all other values of $x_j$ held fixed. The terms $(\hat{y}^{(i)} | X_{\pi ^{B_l}(x_j|\vartheta )}, l)$ and $(\hat{y}^{(i)} | X, l)$ denote the predictions of the true outcome values $y^{(i)}$ made by tree *l*, based on the predictor matrices $X_{\pi ^{B_l}(x_j|\vartheta )}$ and *X*, respectively.

The overall permutation VIMP for variable $x_j$ is then the average over all *q* trees:

5 $$\begin{aligned} VI_j = \frac{1}{q} \sum _{l=1}^{q} VI^{(l)}(x_j). \end{aligned}$$

Since the $VI^{(l)}(x_j)$ values are conditionally independent across trees given the data, the standard error of $VI_j$ can be estimated as $\sigma / \sqrt{q}$, where $\sigma$ is the empirical standard deviation of the $VI^{(l)}(x_j)$ values [18]. This allows computation of a standardized importance score, or z-score, as $VI_j / (\sigma / \sqrt{q})$.

The permutation VIMP is an ad-hoc measure without a known sampling distribution, making formal significance testing nontrivial. Its magnitude also depends on the scale of the outcome variable, which is why there is no general threshold to determine whether a variable is important based on its VIMP score alone.

The next two paragraphs discuss how certain data characteristics, particularly correlated variables and categorical variables, can introduce bias into VIMP scores, potentially affecting the validity of variable selection procedures based on these scores.

***VIMP bias 1: Correlated variables*** A potentially concerning bias of the permutation VIMP was explored by Nicodemus et al. [16], who investigated the behavior of RF permutation VIMP measures in the presence of correlation between variables. Their simulation study involved 12 variables, with four of them strongly correlated to each other. In the scenario in which none of the variables was informative for the outcome, the VIMPs of the four correlated variables were slightly positively biased when the unscaled permutation importance was used. In their study, [16] showed that the bias is notably more pronounced when using the scaled permutation importance, which is the VIMP measure employed in the Boruta algorithm and shadowVIMP. However, shadowVIMP is designed to be robust against this bias by incorporating it into the method’s decision criterion.

***VIMP bias 2: Categorical variables*** Another concerning bias of the permutation VIMP measure is well described by Strobl et al. [3]. Through a simulation study, they demonstrate that for both unscaled and scaled permutation importance in a standard RF, the variance of VIMP scores for uninformative categorical variables increases with the cardinality of the variable. This effect arises from the way categorical variables are handled during tree construction, particularly in the selection of the optimal variable for splitting.

The splitting variable is chosen from the *mtry* candidate variables based on which one selects the best split - defined as minimizing the variance of the outcome within the resulting child nodes. High-cardinality categorical variables provide more potential split points, offering greater flexibility in optimizing this criterion. This increased flexibility raises the likelihood of identifying splits that, by chance, seem to reduce variance, even when the variable is uninformative. As a result, high-cardinality variables are disproportionately frequently selected for splits. Their overrepresentation inflates their influence in the permutation importance calculation, leading to the higher variability of VIMP scores observed by Strobl et al. [3].

In our study, the R package ranger is utilized to construct RFs using the ’order’ method, which handles categorical variables differently from the approach used by Strobl et al. [3]. It involves ordering the categories based on their relationship with the outcome variable once, prior to constructing the forest, using the full data set. Consequently, this ordering, and by extension the splits derived from it, generalize to the OOB data set in its apparent association to the outcome.

When variables whose categories have been reordered using the ’order’ approach are permuted in the OOB data set during the calculation of the permutation importance, the artificially induced relationship between the variable and the outcome (created by the ordering) is disrupted, leading to a drop in accuracy. In our testing we saw that this produces positively biased VIMPs for high-cardinality categorical variables, compounding mechanisms leading to a preference of such variables.

### Existing variable selection approaches

***rfvimptest*** The rfvimptest method [13] closely adheres to the principles of permutation testing. Because the work by Hapfelmeier et al. [13] is closely based on the approach introduced by Hapfelmeie and Ulm [12], we will first present the latter. First, a RF is constructed on the original data set [*y*, *X*] to compute the VIMP score values of all variables. Denote the VIMP score value of the *j*-th variable as $VI_j$. Second, to assess the statistical significance of $VI_j$, a null distribution is generated by permuting $x_j$ in the predictor matrix, yielding a modified data set $[y, X_{\pi (x_j|\vartheta )}]$. A RF is then trained on $[y, X_{\pi (x_j|\vartheta )}]$ and the VIMP score of $\pi (x_j|\vartheta )$ is recomputed. This second step - permuting $x_j$, constructing a RF, calculating the VIMP of $x_j$ - is repeated multiple times, producing a distribution of VIMPs where any association of $x_j$ with the outcome variable is purely by chance. Finally, a p-value is obtained by determining the proportion of iterations in which the VIMP of the permuted variable $\pi (x_j|\vartheta )$ exceeds the original $VI_j$. This proportion provides a statistical measure of whether the observed VIMP score value of $x_j$ could have occurred under the null hypothesis of no association with the outcome. The p-value can then be compared to a chosen significance level $\alpha$ allowing for a final conclusion on whether the variable $x_j$ is considered informative or not. Hapfelmeier et al. [13] extend this approach by introducing a sequential permutation test. This addition allows for early termination of iterations when it becomes clear which hypothesis is most likely to be accepted, thus improving the runtime of the method. The method is implemented in the R package rfvimptest [19] available on CRAN.

***Vita*** The Vita approach is unique among the four methods considered in this publication, as it does not rely on permuting parts of the data set to generate the null distribution for VIMP. Instead, it employs a clever trick: variables with VIMP scores of zero or less are presumed to be uninformative, that is, not associated with the outcome. The distribution of these non-positive VIMPs is then mirrored around zero to construct the full null distribution of VIMPs.

This method relies on the assumption that under the null hypothesis of no association, VIMPs are symmetrically distributed around zero. However, as demonstrated by Janitza et al. [2], this symmetry assumption does not hold for the standard permutation-based VIMP. To address this, the Vita approach uses the hold-out importance, which involves splitting the data set into two equally sized subsets. An RF is trained on one subset while the VIMP is evaluated on the other independent subset, and vice versa. The final importance score is defined as the average.

Once the null distribution is constructed from these hold-out importance scores, determining the critical region for a given significance level becomes straightforward. Variables with VIMPs that fall within this critical region are identified as having a statistically significant influence. One limitation of this process is that it requires a sufficient number of uninformative variables in the data set to provide enough granularity for the mirrored null distribution, thus making it only applicable to high-dimensional data sets.

***Boruta*** The Boruta algorithm extends the predictor matrix by a copy of itself in which all columns (i.e., the variables) have been randomly permuted. Each variable is permuted individually, which breaks the variable’s relationship to the outcome and to all other variables. The permuted variables are referred to as shadow variables as they represent uninformative copies of the original variables. For notation, let *X* be the predictor matrix of the original data set and ${X}_{\pi (x_1,..., x_p|\vartheta _1,..., \vartheta _p)}$ be the matrix of the corresponding individually permuted shadow variables. The extended predictor matrix ${X} \oplus {X}_{\pi (x_1,..., x_p|\vartheta _1,..., \vartheta _p)}$ forms the basis of the Boruta algorithm.

A RF is constructed using the extended predictor matrix, and the scaled permutation VIMP score (or “z-score”) is computed for each variable, including both the original and shadow variables. The maximum z-score among the shadow variables, referred to as MZSA, represents the largest VIMP of variables expected to be uninformative due to the permutation process.

A ’hit’ is assigned to any original variable whose importance score exceeds the MZSA. Since the VIMP scores of both the original and shadow variables can vary due to the randomness of the RF algorithm and the particular realization of the shadow variables, the process of permuting the shadow variables, re-constructing the RF and re-calculating the associated VIMPs is repeated multiple times.

After each iteration, the number of hits each variable scored so far is compared to the expected number of hits using a binomial test, which tests the null hypothesis that a variable’s VIMP is larger than the MZSA just by chance. If a variable scores a VIMP smaller than the MZSA in more than half of the current number of iterations to a statistically significant degree, the variable is deemed uninformative and removed from the data set alongside its shadow variable counterpart. Conversely, if the variable scores a VIMP larger than the MZSA in more than half of the current number of iterations to a statistically significant degree, the variable is deemed informative. Variables for which no statistically significant decision can be reached in the current iteration are categorized as ’tentative’.

The Boruta algorithm iterates until no tentative variables remain or a user-defined maximum number of iterations is reached. Since the statistical test is repeated in each iteration without adjustment for multiplicity across iterations, and the null hypothesis makes the trivially untrue, yet conservative assumption that any single uninformative variable scores VIMPs less than or equal to the maximum of several uninformative shadow variables, this procedure should be viewed as a heuristic method rather than a statistically rigorous approach. We hypothesize that the MZSA cutoff acts as a form of multiplicity adjustment across variables, as the expected value of the MZSA increases with the number of variables in the data set. This implies that the threshold for assigning a ’hit’ in favor of a variable being deemed informative rises with the number of variables and, consequently, the number of hypotheses being tested, ultimately making the procedure more conservative as more variables are evaluated, similar to how multiple testing procedures impose more stringent significance levels as the number of hypotheses increases.

### Proposed new approach: shadowVIMP

#### Permutations and VIMP distributions

Like Boruta, the proposed shadowVIMP method extends the predictor matrix *X* by permuted copies of the original variables. The key difference is in the permutation scheme. While Boruta permutes each variable independently column-wise using ${X}_{\pi (x_1,..., x_p|\vartheta _1,..., \vartheta _p)}$, shadowVIMP instead applies a row-wise permutation, ${X}_{\pi (x_1,..., x_p|\vartheta )}$, meaning that entire rows of *X* are shuffled rather than individual columns. This approach generates ’row-wise permuted’ shadow variables, r-shadows for short. The extended predictor matrix ${X} \oplus {X}_{\pi (x_1,..., x_p|\vartheta )}$ of shadowVIMP has *n* rows and 2*p* columns, the same dimension as the initial version of Boruta at the start of the algorithm.

The motivation behind this permutation strategy is to replicate the correlation structure of the original predictor matrix within the r-shadows. This is important because, as demonstrated by Nicodemus et al. [16], the VIMP of an uninformative variable can be biased upward if it is correlated with other variables. Therefore, for r-shadows to approximate the null distribution - representing the VIMPs the variable would score if it were unrelated to the outcome but all other conditions relevant for VIMP (such as correlations) remained the same - it appears crucial to preserve these correlations in the permutation process. By doing so, a level comparison can be facilitated between the VIMPs of the original variable and its corresponding r-shadow.

In contrast, consider how individual permutations, as used by Boruta and rfvimptest, can lead to misleading results given the findings discussed in section 2.2. By permuting each variable independently, these methods break not only the association between the variable and the outcome but also any correlation between the variable and other variables. Consequently, the VIMPs from the permuted shadow variables become centered around zero. Now once the positively biased VIMP of the original, uninformative and correlated variable is compared against the zero-centered null distribution of the shadow, it will stand out and thus may falsely appear informative. In this case, the observed importance is driven by correlation-induced bias rather than the true relationship with the outcome, potentially leading to false positives.

#### ’Per variable’ decision criterion

The criterion for determining whether a variable is informative is based on a direct comparison between the original variable and its corresponding r-shadow. In each of *niter* iterations, the r-shadows are re-permuted, a RF is constructed, and the VIMP score values for both the original variables and the shadow variables. If $\vec {{VI}}_{j}$ and $\vec {{VI}}^{(r-sha)}_{j}$ represent the vectors of VIMPs scored over niter iterations by the *j*-th original variable and its r-shadow counterpart, the decision is made by comparing the median importance scored by the original variable to the permutation distribution of VIMPs scored by the r-shadow variable. The median VIMP score of the original variable across iterations is used as a robust summary statistic for comparing the importance of the original variable to the VIMP score’s permutation distribution. The decision criterion, given some significance level $\alpha$, is formulated as follows:

6 $$\begin{aligned} {p}_{j} = 1 - \hat{{F}}_{\left\{ \vec {{VI}}^{(r-sha)}_{j}, \ \infty \right\} } (\text {median}(\vec {{VI}}_{j})) \le \alpha \implies {x}_{j} \text { is informative}. \end{aligned}$$

Here, ${p}_{j}$ represents the p-value of variable *j*, testing the hypothesis that the variable has no greater importance than the corresponding r-shadow variable. The addition of $\infty$ as the final element to the vector of shadow VIMPs introduces a small offset to the *p*-values and, most critically, prevents the generation of zero *p*-values, as recommended by Phipson and Smyth [20].

This decision criterion directly compares the original variable to its shadow copy, similar to the rfvimptest method. The key differences include using the median VIMP across iterations of the original variable. Additionally, a different permutation scheme is used which adds the permuted version of the original variable to the set of variables, instead of replacing individual variables with their permuted versions.

In contrast, Boruta’s decision criterion compares each original variable’s VIMP to the maximum VIMP scored across all shadow variables. The concern with this approach is that Boruta combines VIMPs from different shadow variables to set a universal importance cutoff, which becomes problematic when these shadow VIMPs are not comparable. As shown in Strobl et al. [3], the distributions of VIMPs can vary substantially depending on the characteristics of the variables. For instance, continuous and categorical variables often have different VIMP distributions, and categorical variables are further influenced by their cardinality (i.e., the number of levels).

For Boruta, this means that the ’maximum VIMP of shadow variables’ cutoff tends to be dominated by certain variables, such as high-cardinality categorical ones, that naturally score larger VIMPs even when they are unrelated to the outcome. As a result, some truly informative variables might be overlooked because their VIMPs, although positive due to their relationship with the outcome, may be smaller than the inflated cutoff caused by variables with characteristics known to lead to inflated VIMPs.

#### ’Pooled’ decision criterion

The previous section emphasized the importance of one-to-one comparisons, where the VIMP of each original variable is evaluated against the distribution of its own corresponding r-shadow variable. In light of this, it may initially seem surprising that we now introduce a second decision criterion that appears to contradict this principle. This alternative approach involves pooling the VIMPs of all shadow variables and across all iterations to create a single, aggregated pooled r-shadow distribution, which is then used as a common benchmark for testing all variables. Before delving into the technical challenge of how to combine permutation-based VIMP distributions - particularly when the variables differ in characteristics relevant to VIMP - we first outline the motivation for this pooled criterion and discuss its potential advantages.

The motivation behind this pooling approach lies in the challenge of obtaining sufficiently small *p*-values for multiple testing adjustments, while keeping the number of iterations - and thus the overall runtime of the algorithm - reasonable.

In simplified terms, while an unadjusted analysis compares each variable’s p-value to a significance level such as $\alpha = 0.05$, a multiple testing-adjusted analysis compares each p-value to a much smaller significance threshold, shifting the critical region to the far upper tail of the permutation distribution. This essentially necessitates the estimation of very small *p*-values to deem variables as informative.

To better understand the motivation for introducing a pooled decision criterion, consider the granularity of the permutation distribution from which *p*-values are derived. Specifically, under the per-variable decision criterion, the smallest attainable p-value is constrained by the resolution of the empirical distribution $\hat{{F}}_{\{\vec {{VI}}^{(r-sha)}_{j}, \ \infty \}}(.)$, which is limited to 1/(niter+1), where niter denotes the number of permutations or iterations.

For instance, in a data set containing 5,000 variables, applying a Bonferroni or Holm correction to control the family-wise error rate at $\alpha = 0.05$ would require individual *p*-values smaller than 0.05/5000 = 0.00001. Achieving such resolution with the original decision criterion (6) would necessitate at least 100000 iterations. Given that large forests with many variables are already time-consuming to compute, this approach could become impractical for most users.

To address this, the pooled decision criterion is introduced in equation (7). Rather than comparing the original variable’s VIMP solely to the distribution of its corresponding shadow variable, this approach compares it with a pooled distribution of VIMPs from all shadow variables across all iterations. This increases the number of permutation samples to $p \cdot {niter}$ as opposed to only niter.

7 $$\begin{aligned} {p}_{j}^{({pooled)}} = 1 - \hat{{F}}_{\left\{ \widetilde{\vec {{VI}}}^{{(r-sha)}}_1, ..., \widetilde{\vec {{VI}}}^{{(r-sha)}}_{j}, ..., \widetilde{\vec {{VI}}}^{{(r-sha)}}_{p} , \ \infty \right\} }(\text {median}(\widetilde{\vec {{VI}}_{j}})) \le \alpha \implies {x}_{j} \text { is informative}. \end{aligned}$$

with

8 $$\begin{aligned}&\widetilde{\vec {{VI}}}^{{(r-sha})}_{j} = \frac{\vec {{VI}}^{{(r-sha})}_{j} - \text {mean}(\vec {{VI}}^{{(r-sha})}_{j})}{\text {sd}(\vec {{VI}}^{{(r-sha})}_{j})}, \end{aligned}$$

9 $$\begin{aligned}&\widetilde{\vec {{VI}}}_{j} = \frac{\vec {{VI}}_{j} - \text {mean}(\vec {{VI}}^{{(r-sha})}_{j})}{\text {sd}(\vec {{VI}}^{{(r-sha})}_{j})}. \end{aligned}$$

While pooling increases the number of VIMPs used in the comparison, the issue of shadow variables differing in characteristics influencing VIMP scores, such as correlations with other variables or the cardinality of categorical variables still persists. A naive pooling of these VIMP scores would result in the upper quantiles of the pooled shadow distribution being dominated by variables exhibiting the largest bias, whether from strong correlations or high cardinality. To mitigate this, two standardizations are applied.

The first step (8) is to standardize each shadow variable’s VIMPs by subtracting its empirical mean and dividing by its empirical standard deviation, both calculated across the iterations. This ensures that all shadow variables’ VIMP distributions are comparable, with each having the same mean and variance.

The second step (9) adjusts the VIMPs of the original variable accordingly by applying the same transformation using the mean and standard deviation from the corresponding *shadow* variable. While this may seem counterintuitive at first, consider the following: if a shadow variable, which is uninformative by design, consistently exhibits large positive VIMPs (due to bias from factors like correlation or high cardinality), then the threshold for its original counterpart to be considered informative should be adjusted upward accordingly. In the original per-variable decision criterion (6), this was implicitly accounted for, as the upper quantile and the critical region of the shadow distribution would also reflect this bias. In contrast, the pooled criterion relies on a pooled r-shadow distribution that has been standardized to have a mean of zero. Consequently, this bias must now be accounted for explicitly by offsetting the original variable’s VIMPs using (9).

The pooled decision criterion is particularly well-suited for high-dimensional data sets, where computational constraints make per-variable comparisons impractical. Additionally, shadowVIMP offers optional pre-selection steps - described in the next section - to further support analyses in such settings.

#### Pre-selection

Removal of variables during the execution of the algorithm is a key feature of the Boruta method. Kursa and Rudnicki [14] point out that dealing with overly large data sets increases run time substantially and is inconvenient in general. Another stated disadvantage is that many machine-learning algorithms have lower accuracy when the data set contains many irrelevant variables. Regarding Boruta, Kursa and Rudnicki [14] observed that, during the execution of the algorithm, the VIMPs of informative variables tend to increase as uninformative variables are eliminated from the variable set. This leads to a clearer separation between informative and uninformative variables in terms of their VIMPs which in turn results in an increase in sensitivity of correctly classifying informative variables as informative. This observation is beneficial to the task at hand, which aims at separating informative variables from uninformative variables based on their VIMPs.

The shadowVIMP method incorporates this idea of removing uninformative variables early in the process, known as pre-selection. This is optional and, when enabled, involves running the basic algorithm multiple times in succession, with increasingly stricter $\alpha$ thresholds at each stage. Only variables deemed significant in one step proceed to the next, progressively reducing the variable set. For example, if the final $\alpha$ threshold is the conventional 0.05, an initial pre-selection step might use a threshold of 0.3. This would involve running the basic algorithm with fewer iterations using this higher threshold, identifying variables that score in at least the 70th percentile of the shadow distribution. Only the variables confirmed during this step would advance to the next step of the algorithm or an additional pre-selection stage. By default, when pre-selection is enabled, two pre-selection steps are performed: the first with $\alpha = 0.3$, followed by a second with $\alpha = 0.15$. Only the variables surviving both steps are then assessed using the final threshold of $\alpha = 0.05$. Throughout the pre-selection process, the pooled decision criterion is applied.

The basic idea behind this approach is that variables with a sufficiently strong influence to remain significant after multiple testing correction are highly likely to survive the pre-selection steps. Our simulation results in Sect. 3 suggest that this expectation is reasonable.

### Simulation studies

#### Simulation designs

The following introduces four simulation designs taken from different publications related to RF VIMP. For each design, 100 data sets of size 100 were generated in 100 replicates. *Simulation design from *Degenhardt et al. [4] The first simulation design involves a large number of variables, some of them grouped within a block-correlated structure. The design was initially introduced by Chen and Zhang [21] and was used in a modified form in the benchmark study of RF variable selection algorithms by Degenhardt et al. [4]. An implementation of this simulation design in R is available by Szymczak [22].

The outcome depends on three latent variables according to the formula

$$\begin{aligned} {y} = 0.25 \, \exp (4\upsilon _{1}) + \frac{4}{1+\exp (-20(\upsilon _{2}-0.5))} + 3\upsilon _3 + \epsilon \end{aligned}$$

with $\epsilon \sim {N(0, 0.2)}$. The three latent variables $\upsilon _1$, $\upsilon _2$ and $\upsilon _3$, as well as three additional latent variables $\upsilon _4$, $\upsilon _5$ and $\upsilon _6$ are independently sampled from a uniform distribution with support [0, 1]. These six latent variables $\upsilon _{w}, {w}= 1,...,6$, serve as the basis for generating six clusters of correlated variables of size g which enter the model as predictor variables. The correlated predictor variables are generated with the formula

10 $$\begin{aligned} {x}_{{w,j}} = \upsilon _{w} + \left( 0.01 + \frac{0.5({j}-1)}{{g}-1} \right) \cdot \nu , \ \text {where} \ \nu \sim N(0, 0.3), \end{aligned}$$

for ${j} = 1,...,{g}$ and ${w} = 1,...,6$, where ${x}_{{w,j}}$ denotes the *j*-th variable in group *w*. That means, each of the six variables $\upsilon _{w}$, w=1,...,6, out of which only the first three are informative for the outcome, is represented by *g* correlated predictor variables in the predictor matrix. Degenhardt et al. [4] point out that, as *j* increases within a group, the expected correlation of the predictor variable ${x}_{{w,j}}$ to the latent variable $\upsilon _{w}$ decreases. As in Degenhardt et al. [4], two group sizes are considered: 10 and 50. The design with group size 10 then involves 30 informative variables that are block correlated in 3 groups of 10 variables each. Additionally, there are 30 uninformative variables that are block-correlated in the same way. These 60 variables are accompanied by 4940 uninformative noise variables, independently sampled from a uniform distribution with support [0,1]. Similarly, in the design with a group size of 50, there are 150 informative and correlated variables, 150 uninformative and correlated variables, and 4700 independent noise variables. Consequently, both designs comprise a total of 5000 variables.

***Simulation design from*** Friedman [23] The second simulation design includes fewer variables, but incorporates an interaction effect between two of them and a non-linear influence from another. This design was originally introduced by Friedman [23] and has been recently employed in a publication by Hapfelmeier et al. [13] to evaluate multiple VIMP-based variable selection algorithms. This simulation design is implemented in the mlbench package in R [24]. The outcome depends on 5 informative variables according to the formula

$$\begin{aligned} {y} = 10 \sin {(\pi { x}_1{x}_2)} + 20({x}_3-0.5)^2 + 10 {x}_4 + 5 {x}_5 + \epsilon \end{aligned}$$

with $\epsilon \sim {N(0, 0.2)}$. The five informative variables ${x}_1, \dots , {x}_5$, as well as five uninformative variables ${x}_6, \dots , {x}_{10}$ are independently sampled from a uniform distribution with support [0, 1].

**Simulation design from** Strobl et al. [3] The simulation design from Strobl et al. [3] involves categorical variables and was specifically created to illustrate potential biases in VIMP measures. The design consists of five variables that are generated as follows: ${x}_1 \sim {N(0, 1)}$, ${x}_2 \sim {M(2)}$, ${x}_3 \sim {M(4)}$, ${x}_4 \sim {M(10)}$, ${x}_5 \sim {M(20)}$ where M(${k}_{cat}$) stands for the multinomial distribution with equal probabilities for all categorical levels in {0,1,...,${k}_{cat}$-1}. In simple terms, the variables consist of one continuous variable and four categorical variables with differing cardinality between 2 (binary) and 20 levels. The outcome is a binary variable (which makes this prediction task a classification problem) and is simulated with the conditional probabilities ${y}|{x}_2 = 0 \sim {B(0.3)}$ and ${y}|{x}_2 = 1 \sim {B(0.7)}$ with B(.) referring to the Bernoulli distribution. This means that the distribution of the outcome depends only on ${x}_2$, making it an informative variable, while the other variables are uninformative.

***Simulation design from Nicodemus et al. ***[16]*** (Null case)*** The simulation design outlined in Nicodemus et al. [16] involves twelve continuous variables, with four of them strongly correlated, while the other eight are uncorrelated to other variables. For the purpose of this simulation study, we consider the null case, meaning that the outcome is permuted, rendering all variables uninformative. The following equation describes how the outcome is originally generated as a function of informative variables, before permuting the outcome:

$$\begin{aligned} {y} = 5{x}_1&+ 5{x}_2 + 2{x}_3 + 0{x}_4 - 5{x}_5 - 5{x}_6 -2{x}_7 + \epsilon \end{aligned}$$

with $\epsilon \sim {N(0, 0.5)}$. In addition, five further variables not related to the outcome ($x_8, \dots , x_{12}$) are added to the data set. The first four variables (${x}_1, \dots , {x}_4$) are strongly correlated, with a correlation coefficient of 0.9. Thus, the covariance matrix of the twelve variables is given by:

$$\begin{aligned} {Cov(X)} = \begin{pmatrix} 1 & 0.9 & 0.9 & 0.9 & 0 & & 0 \\ 0.9 & 1 & 0.9 & 0.9 & 0 & \dots & 0 \\ 0.9 & 0.9 & 1 & 0.9 & 0 & & 0 \\ 0.9 & 0.9 & 0.9 & 1 & 0 & & 0 \\ 0 & 0 & 0 & 0 & 1 & & 0 \\ & \vdots & & & & \ddots & \\ 0 & 0 & 0 & 0 & 0 & & 1 \\ \end{pmatrix} \end{aligned}$$

#### Benchmark measures

This subsection describes the evaluation metrics used in the simulation study. Each simulation design includes an outcome variable and a mix of informative and/or uninformative predictors; in three of the four designs, the outcome is functionally dependent on the informative variables. In the following, define:

- #Informative: number of variables functionally related to the outcome
- #Uninformative: number of noise variables
- TP (true positives): number of informative variables correctly classified as informative
- FP (false positives): number of uninformative variables wrongly classified as informative

Using these quantities, four measures are calculated for each replicate:

$$\begin{aligned} \text {sensitivity}&= \frac{{TP}}{{\#Informative}} \\ \text {type-1 error rate}&= \frac{{FP}}{{\#Uninformative}} \\ \text {False discovery proportion}&= \frac{{FP}}{{FP} + {TP}}\\ \text {FWER }\ \text { event}&= \mathbf{1}_{\{FP > 0\}} \end{aligned}$$

In the following, the reported quantities are the average (mean) of the above quantities over the 100 replicates. Confidence intervals (95%) are computed by bootstrapping the above quantities 100000 times and calculating the 2.5% and 97.5% quantile. The mean false discovery proportion across iterations and the proportion of iterations with a FWER event serve as estimators of the FDR and FWER, respectively. The results are presented in three parts: (1) an unadjusted analysis comparing sensitivity and type-1 error rates across methods; (2) an FDR-adjusted analysis comparing the sensitivity and FDR of shadowVIMP, rfvimptest, and Vita after applying the Benjamini–Hochberg procedure; and (3) an FWER-adjusted analysis comparing the sensitivity and FWER of the same methods after applying the Holm correction. Note that Boruta does not produce *p*-values and always returns only a binary selection decision. As a result, this method cannot be adjusted for FDR or FWER using standard p-value adjustment methods. However, the unadjusted results are still included in the comparisons of sensitivity against FDR and FWER, though their findings may not be entirely comparable in this context.

#### Configuration of RF and compared variable selection methods

Table 1 provides the configurations used for each method. To ensure a meaningful comparison, key RF parameters–such as the number of trees (num.trees), the mtry parameter, and the handling of categorical variables–are kept consistent across methods, along with the RF implementation itself using the ranger package. Notably, in high-dimensional settings, initial exploratory analyses indicated that increasing num.trees beyond the default value significantly impacted the performance of all methods. Therefore, to maintain consistency, the same sufficiently high num.trees value is used across all methods.

For shadowVIMP, initial testing showed that the pooled decision criterion and the use of pre-selection improved performance in high-dimensional simulation designs. In contrast, for low-dimensional simulation designs, the results were largely consistent regardless of the employed decision criterion (pooled or per-variable) or whether pre-selection was used. Since the pooled decision criterion and the use of pre-selection either performed better or were at least comparable to the alternatives, these settings are recommended as the default configuration for shadowVIMP. Unless otherwise specified, the results are presented using this configuration. Results for alternative configurations of shadowVIMP are presented in Section ?? for the Nicodemus design, and results across all simulation designs can be found in the electronic appendix of this publication [25].

**Table 1:** Summary of configurations of RF and competing variable selection methods

Method	Details
Random forest (ranger)	
num.trees	10000
mtry	$\sqrt{p}$
Importance	permutation: Boruta, rfvimptest, shadowVIMP
	holdout: Vita
Scaled permutation importance	Yes: Boruta, shadowVIMP
	No: rfvimptest, Vita
Handling of categorical variables	respect.unordered.factors = ’ordered’
rfvimptest	type = ’pval’
Vita	ranger::holdoutRF(.) and ranger::importance_pvalues(..., method = ’janitza’)
Boruta	maxRuns = 1200 and Boruta::TentativeRoughFix()
ShadowVIMP	With pre-selection$^a$ and using pooled decision criterion

a: 30 iterations with $\alpha = 0.30$ then 120 iterations with $\alpha = 0.15$ then 1000 iterations with $\alpha = 0.05$. The versions of all R packages used in the simulation study are documented in the GitHub repository $^a$ 30 iterations with $\alpha = 0.30$ then 120 iterations with $\alpha = 0.15$ then 1000 iterations with $\alpha = 0.05$

## Results

The results are presented in Table 2.

**Table 2:** Comparison of sensitivity, type-1 error, FDR, and FWER across different methods and designs

	Unadjusted		FDR-adjusted^a^		FWER-adjusted^a^	
Design Method	Sensitivity [%]	Type-1 error [%]	Sensitivity [%]	FDR [%]	Sensitivity [%]	FWER [%]
**Deg. (10)**						
shadowVIMP	92.2 [90.2, 94.1]	1.9 [1.9, 2.0]	76.3 [74.0, 78.7]	4.6 [3.9, 5.4]	68.1 [67.1, 69.3]	3.0 [0.0, 7.0]
Boruta	70.1 [68.6, 71.8]	0.1 [0.0, 0.1]	70.1 [68.6, 71.8]	11.1 [9.9, 12.2]	70.1 [68.6, 71.8]	95.0 [90.0, 99.0]
Vita	85.5 [83.0, 87.9]	5.2 [5.1, 5.3]	64.0 [61.9, 66.1]	10.3 [8.7, 12.1]	61.2 [58.9, 63.4]	61.0 [51.0, 70.0]
**Deg. (50)**						
shadowVIMP	99.0 [98.5, 99.4]	1.4 [1.3, 1.4]	96.4 [95.4, 97.3]	4.1 [3.7, 4.6]	67.3 [65.4, 69.3]	5.0 [1.0, 10.0]
Boruta	82.6 [80.5, 84.7]	0.0 [0.0, 0.0]	82.6 [80.5, 84.7]	0.8 [0.6, 1.1]	82.6 [80.5, 84.7]	49.0 [39.0, 59.0]
Vita	89.7 [87.6, 91.7]	5.0 [5.0, 5.1]	71.3 [69.7, 73.0]	7.1 [6.4, 7.9]	66.3 [65.2, 67.4]	63.0 [53.0, 72.0]
**Friedman**						
shadowVIMP	96.0 [94.2, 97.6]	4.2 [2.4, 6.2]	93.8 [91.6, 95.8]	2.2 [1.1, 3.4]	89.6 [87.2, 92.0]	6.0 [2.0, 11.0]
Boruta	98.2 [97.0, 99.2]	11.8 [9.2, 14.6]	98.2 [97.0, 99.2]	9.4 [7.4, 11.6]	98.2 [97.0, 99.2]	48.0 [38.0, 58.0]
rfvimptest	95.6 [93.8, 97.2]	5.0 [3.2, 7.2]	93.2 [91.0, 95.2]	2.2 [1.0, 3.5]	88.0 [85.4, 90.6]	7.0 [2.0, 12.0]
**Nico. (Null)**						
shadowVIMP	–	5.6 [4.0, 7.2]	–	6.0 [2.0, 11.0]	–	5.0 [1.0, 10.0]
Boruta	–	17.2 [13.9, 20.7]	–	63.0 [53.0, 72.0]	–	63.0 [53.0, 72.0]
rfvimptest	–	15.1 [12.6, 17.7]	–	14.0 [8.0, 21.0]	–	11.0 [5.0, 17.0]
**Strobl**						
shadowVIMP	96.0 [92.0, 99.0]	3.8 [2.0, 6.0]	92.0 [86.0, 97.0]	4.5 [1.5, 8.0]	92.0 [86.0, 97.0]	5.0 [1.0, 10.0]
Boruta	47.0 [37.0, 57.0]	5.2 [3.0, 7.8]	47.0 [37.0, 57.0]	15.3 [9.0, 22.3]	47.0 [37.0, 57.0]	18.0 [11.0, 26.0]
rfvimptest	92.0 [86.0, 97.0]	5.5 [3.2, 7.8]	82.0 [74.0, 89.0]	6.0 [2.5, 10.0]	82.0 [74.0, 89.0]	7.0 [2.0, 12.0]

Presented is mean and empirical 95% confidence interval of the sensitivity, type-1 error, FDR and FWER across 100 replicates. The FWER is the proportion of replicates with at least 1 false positive. a: FDR- and FWER-adjustment is not applied to Boruta

### Sensitivity, type-1 error rate, FDR, and runtime

This section focuses on those simulation designs that were used to compare the performance of different methods: Degenhardt (10 and 50) and Friedman. These designs provide a general performance comparison, in contrast to designs addressed in later sections (Strobl and Nicodemus) that are specifically designed to illustrate the impacts of biases of the VIMP measure.

For the high-dimensional Degenhardt designs, we focus on FDR-adjusted analyses, evaluating sensitivity and FDR after adjustment. For Friedman, we focus on the unadjusted analysis, comparing the sensitivity and the type-1 error rate.

For the Degenhardt Group Size 10 design, the shadowVIMP method achieved the highest average sensitivity (76.3%) and the lowest FDR (4.6%) among the compared methods. In the Degenhardt Group Size 50 design, shadowVIMP also attained the highest sensitivity (96.4%), with an FDR of 4.1%, reasonably close to the nominal target level of 5%. In the group size 50 design, the Boruta method had the lowest FDR (0.8%) among the compared methods but exhibited considerably lower sensitivity compared to shadowVIMP (82.6% vs. 96.4%). Vita showed the lowest sensitivity after FDR adjustment among the methods for both high-dimensional designs.

The results of Boruta and Vita in the two high-dimensional designs are largely consistent with the findings presented in the paper by Degenhardt et al. [4]. However, a direct comparison is challenging due to differences in the FDR adjustment method and reporting of the median result across replicates in the previous publication versus the mean in the current study.

For the low-dimensional performance benchmark (Friedman design), shadowVIMP, Boruta, and rfvimptest achieved comparable sensitivities. However, Boruta exhibited a noticeably higher type-1 error rate (11.8%) compared to shadowVIMP (4.2%) and rfvimptest (5.0%).

For FDR- and FWER-adjusted analyses on the Friedman design, shadowVIMP and rfvimptest demonstrated nearly identical performance.

The runtime of each method varies depending on factors such as the number of trees, iterations, method-specific parameters, and the characteristics of the data set. Although not representing an extensive runtime comparison, the following comparisons provide useful insight into runtime expectations. For the high-dimensional Degenhardt Group Size 50 design, shadowVIMP completed in approximately 20 min, while Boruta, with its early stopping mechanism, took 1 to 15 min depending on the replicate. Vita, which does not rely on iterations, concluded in under 1 min. For the small-scale Friedman design, shadowVIMP took approximately 5 min, reflecting a likely excessive number of iterations, while Boruta ranged from just seconds to 5 min depending on when the early stopping occurred. The rfvimptest method exhibited some runtime variation across replicates but completed within 5 min.

### Evaluation of nominal significance level adherence

This section investigates and compares the four methods with respect to the adherence to the nominal significance level. The comparison examines the type-1 error rate, the FDR, and the FWER, the nominal (targeted) level being the conventional 5%. Although the Boruta method allows specifying a significance level as a parameter in its function call, it does not explicitly control for a nominal significance level in the same way as the other methods. Nevertheless, we include its results for comparability.

The results are shown in Table 2. In all five considered designs, the shadowVIMP method demonstrates a type-1 error rate and the FDR (after Benjamini-Hochberg adjustment) at or below the nominal significance level of 5%. A minor exception is the type-1 error rate and FDR for the Nicodemus design at 5.6%, and 6%, respectively. For the FWER, after applying the Holm-Bonferroni adjustment, the shadowVIMP method shows a FWER of 5% or less in 4 out of 5 designs, and 6% for the Friedman design. With each design evaluated using 100 replicates, a 5% FWER indicates a false positive in 5 out of 100 replicates. Due to the limited number of replicates, the 95% empirical confidence intervals around the FWER estimates are large, ranging from 0 to 11%.

The Vita method, applicable to high-dimensional data sets such as Degenhardt Group Size 10 and 50 designs, displays type-1 error rates of 5.2% and 5%, validating the method in this regard. The FDR, after Benjamini-Hochberg adjustment, is 10.3% and 7.1%, respectively. Multiple testing adjustment was not discussed in the original publication, so these results should be interpreted as exploratory. The FWER after Holm’s adjustment is 61.2% and 66.3%, suggesting that the FWER adjustment does not perform well in our simulation study in the case of the Vita method.

The rfvimptest method is applied only for the low-dimensional designs Friedman, Nicodemus, and Strobl. The results show a type-1 error rate of 5.0% for the Friedman design and 5.5% for the Strobl design, close to the targeted nominal level of 5%. In stark contrast, for the Nicodemus design, rfvimptest displayed a type-1 error rate of 15.1%, likely due to biases in the VIMP measure with respect to correlated variables. This result is further discussed in section 3.3.

Finally, the results of the Boruta method differ substantially across the five designs. The type-1 error rate ranges from less than 0.1% in high-dimensional designs to 17.2% for the Nicodemus design. Without applying multiple testing adjustments, the FDR varies considerably between the high-dimensional designs (0.8% for Group Size 50 and 11.1% for Group Size 10). The FDR for the Friedman and Strobl designs is 9.4% and 15.3%, respectively, and for the Nicodemus design, it is 63%. Despite mentions of a ’multiple comparison adjustment’ in Boruta’s documentation in the form of Bonferroni’s FWER adjustment, its repeated testing on the same results means it cannot be expected to hold any specified nominal significance level. The realized FWERs across the five different simulation designs reflect this, as they are between 18% and 95%.

### VIMP bias 1: correlated variables

Boruta and rfvimptest exhibit higher type-1 error rates in the Nicodemus scenario with uninformative correlated variables, most likely because they individually permute columns, thereby disrupting the correlation structure present among the original variables. The presence or absence of correlations between variables is particularly important in this context because previous research by Nicodemus et al. [16] demonstrated a bias of the VIMP measure of correlated variables when using the permutation importance measure with RFs employing CART trees. For Boruta and rfvimptest, the disruption of the correlation structure leads to a situation where biased VIMP scores of the original uninformative variables are compared against shadow variables whose VIMP scores have zero mean, which can lead to false identification of the original variables as informative. For example, as presented in Table 3, Boruta exhibits a false selection proportion as high as 37% for the correlated variables $x_1, \dots , x_4$, resulting in an overall average type-1 error rate of 17.2%. Similarly, rfvimptest shows a false selection proportion of 32% for the correlated variables and 6.6% for the uncorrelated variables $x_5, \dots , x_{12}$, leading to an overall average type-1 error rate of 15.1%.

In contrast, as detailed in section 2.4, shadowVIMP addresses this issue by constructing r-shadow variables that maintain the exact correlation structure of the original variables allowing for a comparison on an even playing field. As a result, shadowVIMP effectively increases the threshold for deeming a correlated variable important in terms of absolute VIMP. In the simulation study, it achieved the intended effect, demonstrating robustness against correlation-induced bias and keeping the type-1 error rate close to the nominal level of 5%. The shadowVIMP false selection proportion for individual variables ranges from 2 to 8%, with no apparent differences between the correlated and uncorrelated variables.

**Table 3:** False selection proportions of each variable across replicates of the Nicodemus design (null case) across different methods

Method	False selection proportion by variable [%]												
	Correlated variables				Uncorrelated variables								
	X1	X2	X3	X4	X5	X6	X7	X8	X9	X10	X11	X12	Type-1 error rate [%]
Boruta	35	34	34	37	8	7	10	5	10	9	11	7	17.2
rfvimptest	29	34	27	38	6	6	9	4	6	8	7	7	15.1
shadowVIMP^a^	3	8	6	6	5	6	6	2	5	8	7	5	5.6
shadowVIMP^b^	5	2	4	3	6	5	6	4	7	7	8	5	5.2
shadowVIMP^c^	3	7	6	6	5	4	6	2	5	7	7	5	5.3
shadowVIMP^d^	6	3	5	4	5	4	6	4	7	7	8	5	5.3

Results for different configurations of shadowVIMP are presented: a: pooled decision criterion and using pre-selection; b: pooled decision criterion and not using pre-selection; c: per variable decision criterion and using pre-selection; d: per variable decision criterion and not using pre-selection

### VIMP bias 2: categorical variables

When evaluating VIMPs in data sets that include categorical variables, special care must be taken. Previous research by Strobl et al. [3] demonstrated through simulation studies that the distribution of VIMPs for a categorical variable is strongly influenced by its cardinality and can differ from continuous variables. Although the method of handling categorical variables in this publication differs in some aspects from the methods used in the original study, as detailed in section 2.2, these findings were replicated using ranger’s ’order’ method applied here.

Given these insights, it appears crucial to compare the VIMP of a variable with the distribution of VIMPs from a shadow variable with the same characteristics as the original variable to ensure sensible comparisons. For instance, a continuous variable should be compared to a continuous shadow variable, and a categorical variable should be compared to a categorical shadow variable with the same cardinality. Following this reasoning, it was expected that the Boruta algorithm might not perform well for the design by Strobl et al. [3] which involves both continuous variables and categorical variables of varying cardinality. This is because Boruta compares the VIMPs of original variables to the maximum VIMPs across all shadow variables, a comparison that is likely to be biased towards the shadow variable with the highest cardinality (e.g., the one with 20 categories).

The results given in Table 2 indicate that this expectation may be true, showing that Boruta’s sensitivity is 47%, much lower than the 92% sensitivity of rfvimptest and the 96% sensitivity of shadowVIMP. Boruta’s lower sensitivity is likely due to the fact that, although the VIMPs of the single informative variable in the design are positive and clearly stand out, there are many instances in which they are still lower than the bias of the high-cardinality shadow variable they are compared to. In contrast, rfvimptest performs ’1:1’ comparisons - comparing each variable directly to its corresponding permutated counterpart - and thus remains unaffected by this issue.

For shadowVIMP, there are two decision criteria. The first, per variable, is a straightforward 1:1 comparison and, as expected, is unaffected by these issues. The second criterion, ’pooled’, compares the original variable to a pooled distribution across all r-shadow variables, and thus it appeared important to investigate whether it would lead to the same issues observed with Boruta. However, the results indicate that the pooled criterion effectively handles these issues well due to the standardization process (see section 2.4) in which the original variable is scaled and centered according to the distribution of its corresponding r-shadow variable, which effectively retains the characteristic of a 1:1 comparison.

In summary, both rfvimptest and shadowVIMP perform well in this design, while the results of Boruta suggest that caution may be needed when applying it to data sets with categorical variables. Different configurations of shadowVIMP produced similar results, regardless of whether pre-selection was used or whether the per-variable or pooled criterion was applied.

### Practical example: Alzheimer’s disease

This section offers a practical example demonstrating the application of shadowVIMP with the aim of showcasing how its visual output can effectively guide researchers by providing different sets of selected variables, depending on the type of multiple testing adjustment required. It is based on a version of the data set from Craig-Schapiro et al. [8]’s study on Alzheimer’s disease, as provided in the AppliedPredictiveModeling R package [9]. According to the package documentation, this is a modified version of the original data set. As such, the results are not directly comparable to those of the original study. The modified data set contains 333 individuals and 130 variables, both continuous and categorical. The outcome is binary and differentiates between cognitively normal and cognitively impaired individuals.

**Fig. 2:**
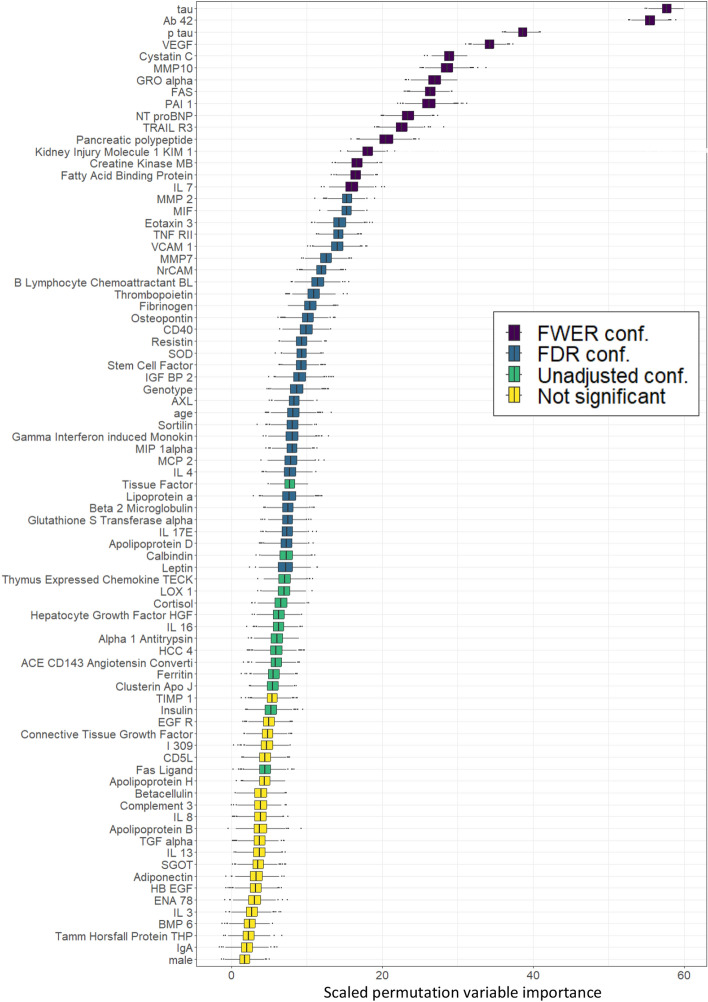
Visual output of the final step of shadowVIMP on the Alzheimer’s disease data set. Two pre-selection steps were applied beforehand and the results are based on the pooled decision criterion. The boxplots display the VIMPs scored by each biomarker across 1000 RF iterations. Each biomarker is colored according to the multiple testing threshold at which the biomarker would be selected

The shadowVIMP method is applied with two pre-selection steps with 30 and 120 RF iterations and the corresponding $\alpha$ thresholds of 0.3 and 0.15, respectively. The results are based on the pooled decision criterion with 1000 RF iterations and a significance level set to $\alpha = 0.05$. They are shown in Fig. 2.

The number of biomarkers selected by the proposed method after Holm’s FWER adjustment is 16. All biomarkers confirmed under Holm’s adjustment also pass Benjamini-Hochberg’s FDR adjustment. An additional 30 biomarkers are considered significant under the FDR adjustment, resulting in 46 biomarkers identified as significant using this approach. Without any multiple testing adjustments, 14 additional biomarkers would be selected.

In Fig. 2, only the biomarkers that pass both pre-selection steps are displayed. Initially, there are 130 biomarkers under consideration. However, in the first pre-selection step, 29 are excluded because they delivered *p*-values greater than 0.3. Following this, the second pre-selection step results in an additional 20 biomarkers being excluded, as they had *p*-values greater than 0.15. Consequently, the figure features only the 81 remaining biomarkers that are present in the final step of the shadowVIMP method.

It can be observed that the VIMPs of individual biomarkers exhibit non-negligible variation across 1,000 RF iterations, each consisting of 10,000 trees. Although further increasing the number of trees would reduce randomness, it is worth noting that even at 10,000 trees, the extent of variation remains substantial. In a scenario where variable selection is based on the top-N variables from a single RF run, this variation could lead to instability in the selected features, as the ranking of the highest-scoring VIMPs fluctuates between iterations. This underscores the usefulness of conducting multiple RF iterations to reveal and mitigate the impact of randomness inherent to the RF classifier.

In shadowVIMP, each biomarker’s median VIMP is either adjusted based on the corresponding r-shadow VIMP distribution (pooled; used here) or directly compared to its r-shadow (per-variable). Consequently, a variable’s group membership–such as inclusion in the “FDR confirmed” category–is not determined solely by the absolute value of its median VIMP. It is determined in relation to the VIMP distribution of the corresponding r-shadow variable. If the distributions of VIMPs of the corresponding r-shadow variables (i.e. the null distributions) vary, a variable with a relatively low median VIMP can achieve a significant p-value, even though there are variables with higher absolute median VIMPs which are classified as insignificant.

## Discussion

In the simulation study, the shadowVIMP method - employing the pooled decision criterion and pre-selection - demonstrated superior sensitivity compared to Boruta and Vita in correctly detecting truly informative variables in the high-dimensional designs used by Degenhardt et al. [4]. The rfvimptest method was not applied to these designs due to its computational requirements increasing with the number of variables. In the smaller simulation design by Friedman [23], shadowVIMP, Boruta, and rfvimptest showed comparable sensitivity. However, Boruta exhibited a type-1 error rate twice as high as shadowVIMP and rfvimptest.

The combination of shadowVIMP’s decision critera taking into account the permutation distribution of the corresponding r-shadow variables combined with its row-wise permutation scheme - which preserves correlations - made it resilient to biased VIMP results related to correlated and categorical variables. This was demonstrated using the simulation designs of Strobl et al. [3] and Nicodemus et al. [16]. In contrast, Boruta was directly and adversely affected by these biases. Similarly, rfvimptest, using CART-based VIMPs, showed susceptibility to correlation bias, leading to inflated type-1 error rates for highly correlated variables. One potential avenue to remedy this issue for rfvimptest is the use of conditional permutation importance using conditional inference forests. This approach was shown to be less prone to correlation bias in the original study by Nicodemus et al. [16], and may offer a way to address the issue at its root by mitigating bias in the underlying VIMPs of correlated variables. However, conditional inference forests are considerably more computationally intensive than standard random forests, which would be a disadvantage in this context, especially given the relatively large numbers of forests that must be built in the case of rfvimptest.

To more clearly demonstrate the specific benefit of the row-wise permutation approach employed by shadowVIMP, we further investigated its effect in additional simulations. Although Boruta’s inflated false selection rates for correlated variables on the Nicodemus design compared to shadowVIMP’s non-inflated rates (Sect. 3.3) already suggest an advantage of the row-wise approach, this comparison does not fully isolate the effect, as Boruta also differs from shadowVIMP in other aspects. To address this, we conducted additional simulations, presented in Appendix 4 and 5, directly comparing shadowVIMP in its original row-wise permutation form against a modified version using individual column permutations to create the shadow variables. These results indicate that the row-wise permutation approach yields modest performance gains and is crucial to avoid the correlation-induced bias described above.

The Benjamini-Hochberg procedure for FDR control and the Holm procedure for FWER control are common basic approaches and are used here for practicality. It should be noted that, depending on the context—such as prior information [26], strongly correlated variables, or subgroup structures [27]—alternative multiple-testing methods may offer improved power.

A key advantage of shadowVIMP over the competing methods is its ability to provide multiple testing-adjusted results, even for high-dimensional data sets. Multiple testing adjustments in such contexts are challenging due to the need for very small *p*-values, which are constrained by the number of shadow VIMPs and thus by the number of iterations performed. While rfvimptest collects only one VIMP per iteration to inform decisions, both shadowVIMP and Boruta are more efficient in this regard by appending the full predictor matrix to the original data during each iteration and using all estimated VIMPs. However, Boruta’s decision rule–deeming a variable informative if its VIMP consistently exceeds the maximum VIMP of shadow variables–is data set-dependent and can be overly strict in high-dimensional settings (type-1 error rates < 0.1%) and too lenient in low-dimensional settings (type-1 error rates > 10%). In contrast, shadowVIMP decision criteria align more closely with frequentist principles by explicitly controlling significance levels, rather than relying on a heuristic rule-based approach.

A strength of this work is the usage of simulation designs previously employed in key literature on RFs and VIMP measures [3, 4, 13, 16].

The shadowVIMP method exhibited higher, but still reasonable, runtimes - around 20 min for the high-dimensional design, and less for the low-dimensional designs. Boruta and rfvimptest benefit from early stopping mechanisms, while Vita is nearly instantaneous, as it does not rely on permutations. However, in practical applications, the time spent analyzing the data is often minor compared to the time required for data collection and preprocessing.

A current limitation of shadowVIMP is the lack of a universal recommendation for the optimal number of iterations. The appropriate number likely depends on the number of variables and whether multiple testing adjustments are applied. A practical starting point is to calculate the minimum number of iterations needed to yield the smallest required p-value. Future research could refine this by developing clearer guidelines or implementing a stability-based stopping criterion, similar to rfvimptest, to optimize iteration counts while maintaining valid results. This would be a manageable scope for a student’s thesis and could be a valuable practical contribution.

In high-dimensional settings, the permutation-based *p*-values obtained with shadowVIMP deviate from the uniform distribution under the null hypothesis (results not shown). This deviation arises from residual random forest stochasticity that remains in the VIMPs of the r-shadow variables but is suppressed in the corresponding VIMPs of the original variables, since their VIMPs are aggregated across iterations by taking the median. Consequently, the null distribution is somewhat broader, leading to conservative *p*-values and a type-1 error rate at or below the nominal level. We view this conservativeness as a robustness property that is particularly desirable in light of the ongoing replication crisis, as it reduces the risk of spurious findings.

## Conclusion

This work introduced shadowVIMP, a variable selection method based on RF that enables multiple testing-adjusted inference for both low- and high-dimensional data while addressing known biases in VIMP caused by categorical or correlated variables. Simulation results using four established designs from the RF and VIMP literature indicated that shadowVIMP maintains the nominal significance level and performs well in terms of power in both low- and high-dimensional settings. A practical example demonstrated how standard RF plots can be extended with information on the statistical significance of the variables, both with and without adjusting for multiple testing, providing a statistically guided framework to support variable selection. The shadowVIMP method is available as an R package shadowVIMP on CRAN [28].

## Data Availability

All materials are available on the following GitHub repository: https://github.com/mueller-staburo/shadowVIMP-Publication.

## Appendix A Supplementary results: comparing row-wise vs. individual column permutation approaches in shadowVIMP

Tables 4 and 5 present additional analyses comparing shadowVIMP in its original row-wise permutation form against a modified version using individual column permutations (Boruta-like). Table 4 reports performance metrics including sensitivity and type-1 error for the Nicodemus (group size 50) and the Nicodemus (Null) design, while Table 5 shows by-variable false selection proportions in the Nicodemus (Null) design.

**Table 4:** Comparison of shadowVIMP using (a) its original row-wise permutation scheme to construct shadow variables and (b) a modified version using individual column permutations (Boruta-like)

	Unadjusted		FDR-adjusted		FWER-adjusted	
Design						
Method	Sensitivity [%]	Type-1 error [%]	Sensitivity [%]	FDR [%]	Sensitivity [%]	FWER [%]
Deg. (50)						
shadowVIMP						
– Original^a^	99.0 [98.5, 99.4]	1.4 [1.3, 1.4]	96.4 [95.4, 97.3]	4.1 [3.7, 4.6]	67.3 [65.4, 69.3]	5.0 [1.0, 10.0]
– Modified^b^	99.0 [98.5, 99.4]	1.3 [1.3, 1.4]	93.4 [91.9, 94.9]	3.1 [2.7, 3.4]	59.1 [57.3, 61.0]	3.0 [0.0, 7.0]
Nico. (Null)						
shadowVIMP						
– Original^a^	–	5.6 [4.0, 7.2]	–	6.0 [2.0, 11.0]	–	5.0 [1.0, 10.0]
– Modified^b^	–	14.2 [11.8, 16.8]	–	9.0 [4.0, 15.0]	–	7.0 [2.0, 12.0]

Values are means with empirical 95% confidence intervals over 100 replicates for two correlated-variable designs. The Nicodemus (Null) design has no informative variables; sensitivity is not reported

**Table 5:** Comparison of shadowVIMP using (a) its original row-wise permutation scheme to construct shadow variables and (b) a modified version using individual column permutations (Boruta-like)

Design/Method	False selection proportion by variable [%]												Type-1 error rate [%]
	Correlated variables				Uncorrelated variables								
	X1	X2	X3	X4	X5	X6	X7	X8	X9	X10	X11	X12	
Nicodemus (Null)													
shadowVIMP													
– Original^a^	3	8	6	6	5	6	6	2	5	8	7	5	5.6
– Modified^b^	27	35	38	36	2	6	5	4	4	2	5	7	14.3

Presented are by-variable false selection proportions in the Nicodemus (Null) design. Values are percentages of false selections across replicates, with the overall Type-1 error rate reported in the final column
